# Genetically proxied IL-1 receptor antagonism and risk of polymyalgia rheumatica

**DOI:** 10.1093/rheumatology/kead423

**Published:** 2023-08-18

**Authors:** Sizheng Steven Zhao, Thurkka Rajeswaran, Sarah L Mackie, James Yarmolinsky

**Affiliations:** Centre for Epidemiology Versus Arthritis, Division of Musculoskeletal and Dermatological Science, School of Biological Sciences, Faculty of Biological Medicine and Health, The University of Manchester, Manchester Academic Health Science Centre, Manchester, UK; Leeds Institute of Rheumatic and Musculoskeletal Medicine, University of Leeds, Leeds, UK; Leeds Institute of Rheumatic and Musculoskeletal Medicine, University of Leeds, Leeds, UK; National Institute for Health Research Leeds Biomedical Research Centre, Leeds Teaching Hospitals, University of Leeds, Leeds, UK; MRC Integrative Epidemiology Unit, University of Bristol, Bristol, UK; Population Health Sciences, Bristol Medical School, University of Bristol, Bristol, UK

Rheumatology key message

- IL-1 receptor antagonism is associated with reduced risk of PMR.

Dear Editor, PMR is a symmetrical, glucocorticoid-sensitive, inflammatory disorder of extracapsular structures [1]. PMR affects chronically mechanically stressed fibrocartilage-containing structures such as sternoclavicular joints, pubic symphysis, entheses and interspinous ligaments. IL-1 generated by activation of the NLRP3 inflammasome, from autoinflammatory stimuli such as calcium pyrophosphate crystals that are commonly found in fibrocartilage, might then be amplified locally by IL-6 and spread contralaterally [2]. Compared with glucocorticoids, the disadvantages of IL-6 inhibition as a therapeutic strategy for PMR include slow onset of benefit and long half-life for washout in the case of adverse effects. An alternative strategy for PMR relapse prevention might be to inhibit IL-1.

The potential efficacy of pharmacologically targeting a protein can be investigated by leveraging naturally occurring genetic variation that mimics its perturbation. Drugs supported by human genetic data are over twice as likely to receive regulatory approval and this approach correctly predicted clinical trial success of IL-6 receptor inhibition for PMR [3]. We examined the potential effect of genetically proxied IL-1 receptor antagonist (IL-1Ra) on risk of PMR.

IL-1Ra is a naturally occurring competitive inhibitor of IL-1; anakinra—a recombinant IL-1Ra—is approved for another immune-mediated inflammatory disease, RA. We proxied IL-1Ra using two complementary approaches. First, we selected weakly correlated (r^2^ < 0.1 using the 1000 Genomes Project as reference panel) single-nucleotide polymorphisms (SNPs) within or ±50 kilobases from the *IL1RN* gene that were associated with circulating CRP (natural-log transformed mg/l) at genome-wide significance (*P* < 5 × 10^−8^) from a genome-wide association study (GWAS) of 204 402 individuals of European ancestry [4]. We selected CRP as the biomarker because IL-1 inhibition reduces CRP concentrations [5]. Second, we applied the same instrument selection process to genetic data for circulating IL-1Ra (i.e. cis-protein quantitative trait loci), from a GWAS meta-analysis of 55 792 individuals of European ancestry [6]. We evaluated instrument strength by calculating F-statistics estimated using beta^2^/s.e.^2^ (F > 10 suggests low likelihood of weak instrument bias). Genetic data for PMR were obtained from a GWAS of 4285 cases and 17 140 controls of self-reported white British ancestry in the UK Biobank [3]. We included RA (22 350 cases; 74 823 controls [7]) as a positive control outcome to examine instrument validity. We used the inverse-variance weighted method and accounted for weak linkage disequilibrium. Pair-wise conditional colocalization was performed to investigate potential genetic confounding through linkage disequilibrium. We used a curated genotype–phenotype database PhenoScanner to search for associations between variants used to instrument each drug target and other traits that may represent pleiotropic pathways. Further information on methodological approaches employed are provided in Supplementary materials.

Using CRP as the biomarker, three SNPs were selected to proxy IL-1Ra signalling (median F-statistic 69) (Supplementary Table S1, available at *Rheumatology* online). Genetically proxied IL-1Ra was associated with a reduced risk of PMR [odds ratio 0.40 per log(mg/l) reduction in CRP; 95% CI 0.16, 0.97; *P* = 0.04]. Results were concordant when using nine variants (median F-statistic 81) to instrument circulating IL-1Ra (odds ratio 0.79 per s.d. increase in IL-1Ra; 95% CI 0.63, 1.00; *P* = 0.051). Results across both analyses were not driven by any single SNP (Supplementary Fig. S1). Colocalization analysis was underpowered, but showed low posterior probabilities of both genetic confounding and the presence of causal variants in *IL1RN* (Supplementary Fig. S2 and Table S2). IL-1Ra was associated with reduced risk of RA (Fig. 1). We found no evidence that the genetic instruments used to proxy IL-1Ra were associated with expression of other cytokines that may represent pleiotropic pathways.

**Figure 1.**
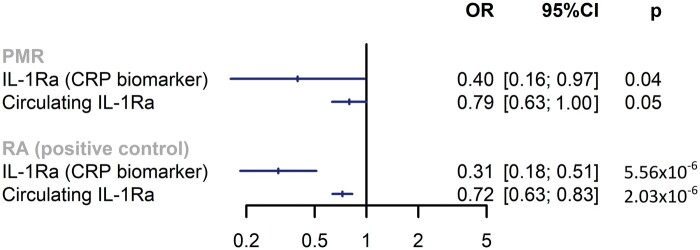
Genetically proxied IL-1 receptor antagonism using CRP as downstream biomarker and circulating IL-1Ra levels. Estimates are scaled to per unit reduction in natural-log transformed mg/l and per s.d. increase in circulating IL-1Ra level

This study provides genetic evidence that IL-1Ra is causally associated with reduced risk of PMR, which was consistent across two approaches to proxy the drug target. These findings are consistent with a small candidate gene study (*n* = 139) that suggested a potential role for *IL1RN* in PMR susceptibility [8], and supports the role of IL-1 signalling in PMR pathogenesis. Long-term glucocorticoids remain the cornerstone of PMR treatment, which can lead to significant additional morbidity in typically elderly and often comorbid individuals. Steroid-sparing treatment options are limited. These results support IL-1Ra, such as anakinra, as a potential therapeutic candidate. However, these estimates may not be directly comparable to pharmacological inhibition because life-long drug target perturbation proxied by germline genetic variants differs from shorter duration of clinical interventions. Our findings relate to risk rather than treatment of PMR, although these are likely to coincide as is the case for IL-6 receptor inhibition and PMR. Clinical studies are needed to test IL-1 inhibition as a therapeutic strategy for PMR.

## Supplementary Material

kead423_Supplementary_Data

## Data Availability

UK Biobank data are available to all bona fide researchers for use in health-related research that is in the public interest. The application procedure is described at www.ukbiobank.ac.uk.
