# Supplementary material for: Genetically proxied IL-1 receptor antagonism and risk of polymyalgia rheumatica
Source: Rheumatology (Oxford). 2023 Aug 18;63(2):e43–4. doi: 10.1093/rheumatology/kead423 (PMC10836967; doi:10.1093/rheumatology/kead423)
Supplement: kead423_Supplementary_Data [file kead423_supplementary_data.docx]

**Supplementary materials for**

**Genetically proxied IL-1 receptor antagonism and risk of polymyalgia rheumatica**

Sizheng Steven Zhao^1^, Thurkka Rajeswaran^2^, Sarah L Mackie^2,3^, James Yarmolinsky^4,5^

1 Centre for Epidemiology Versus Arthritis, Division of Musculoskeletal and Dermatological Science, School of Biological Sciences, Faculty of Biological Medicine and Health, The University of Manchester, Manchester Academic Health Science Centre, Manchester, UK

2 Leeds Institute of Rheumatic and Musculoskeletal Medicine, University of Leeds, Leeds, UK

3 National Institute for Health Research Leeds Biomedical Research Centre, Leeds Teaching Hospitals, University of Leeds, Leeds, UK

4 MRC Integrative Epidemiology Unit, University of Bristol, Bristol, UK

5 Population Health Sciences, Bristol Medical School, University of Bristol, Bristol, UK

Correspondence to: Sizheng Steven Zhao. Centre for Epidemiology Versus Arthritis, Division of Musculoskeletal and Dermatological Science, School of Biological Sciences, Faculty of Biological Medicine and Health, The University of Manchester, Manchester Academic Health Science Centre, Oxford Road, Manchester, M13 9LJ, UK. Email: [Sizheng.zhao@manchester.ac.uk](mailto:Sizheng.zhao@manchester.ac.uk)

Contents

[Supplementary Methods 2](#_Toc138073218)

[Figure S1. Leave-one-out Mendelian randomization analysis. 3](#_Toc138073219)

[Figure S2. Locus plots of PMR, CRP and IL-1Ra in and ± 50kilobases from the *IL1RN* gene region. 4](#_Toc138073220)

[Table S1. Single-nucleotide polymorphisms use to instrument IL-1Ra. 5](#_Toc138073221)

[Table S2. Colocalization analysis results. 6](#_Toc138073222)

[References 7](#_Toc138073223)

# Supplementary Methods

Mendelian randomization (MR) can be conceptualised as a quasi-randomised (because genetic variants are randomly assorted at meiosis within each individual) natural experiment that is typically more robust against biases pervasive in traditional epidemiologic designs, such as confounding and reverse causation [1].

A genetic variant can be considered as a valid instrumental variable for an exposure if it satisfies the instrumental variable assumptions: it is associated with the exposure (assumption 1), it is not associated with the outcome due to confounding (assumption 2), and it does not affect the outcome except via the exposure (assumption 3).


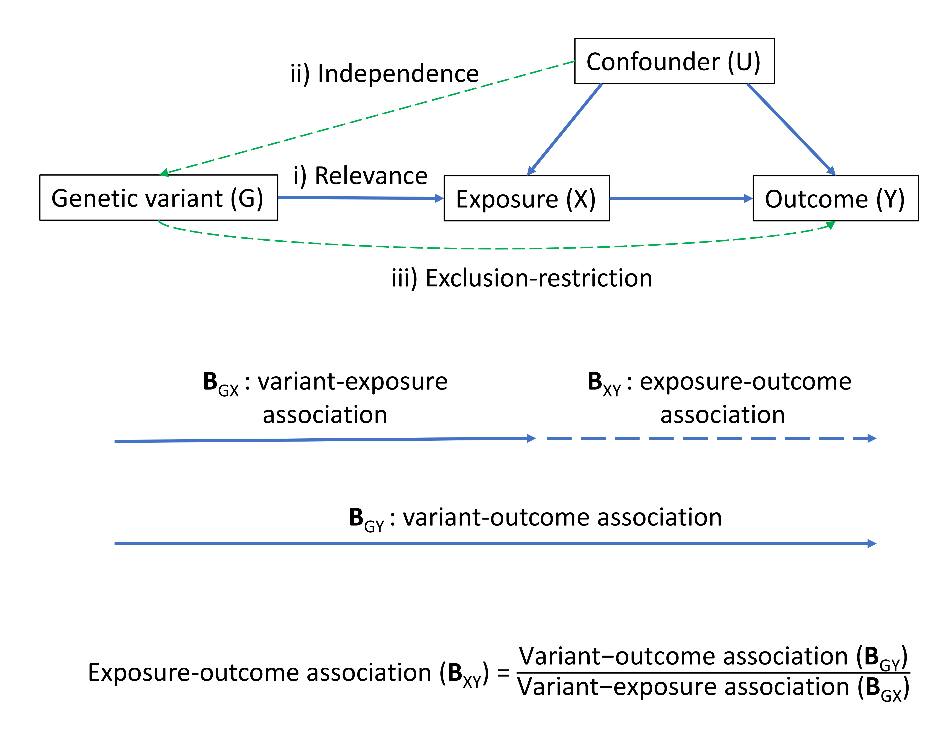


To maximize statistical power of our analyses, we selected instruments that were permitted to be weakly correlated with each other (i.e., in linkage disequilibrium (LD)) [2]. Analyses accounted for LD pattern between the SNPs, where the pairwise r^2^ values were obtained from the 1000 Genomes European ancestry reference panel. Analyses were performed using the *MendelianRandomization* package in R [3].

MR associations can arise from genetic confounding due to LD. We performed colocalization analysis to examine this potential source of bias, using GCTA-COJO and the coloc package as implemented in Pair-Wise Conditional Colocalisation analysis (PWCoCo) [4]. We used default prior probabilities that any SNP within the colocalization window (± 50kilobases from *IL1RN*) was associated exclusively with the exposure (p_1_=1x10^-4^), exclusively with PMR (p_2_=1x10^-4^), or both (p_12_=1x10^-5^).


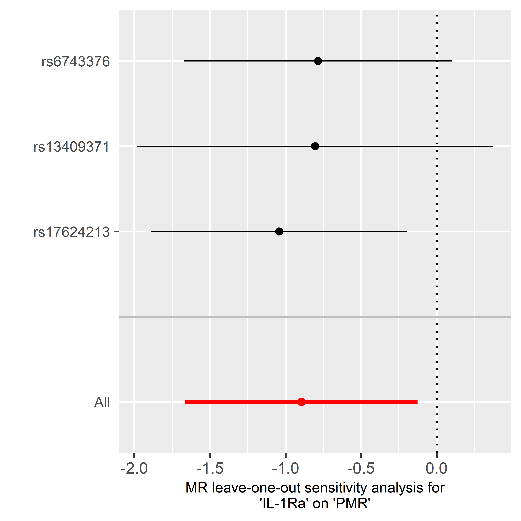

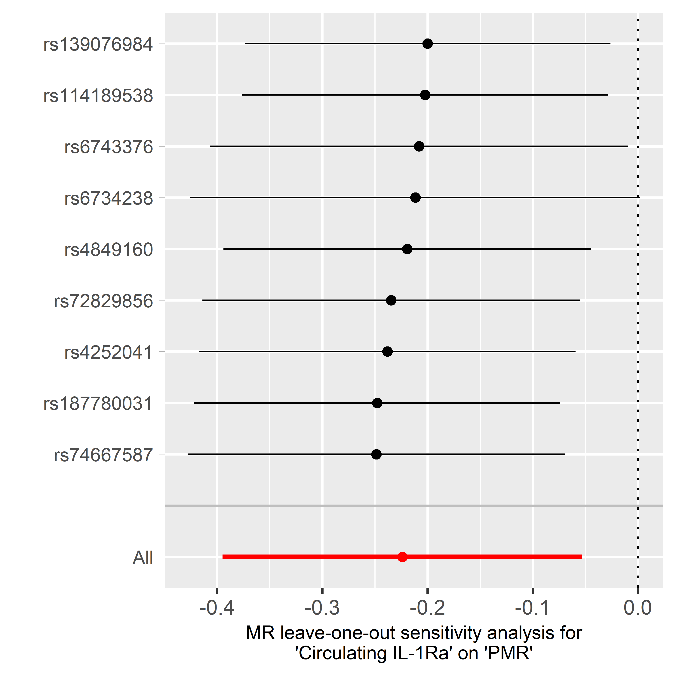


# Figure S1. Leave-one-out Mendelian randomization analysis.


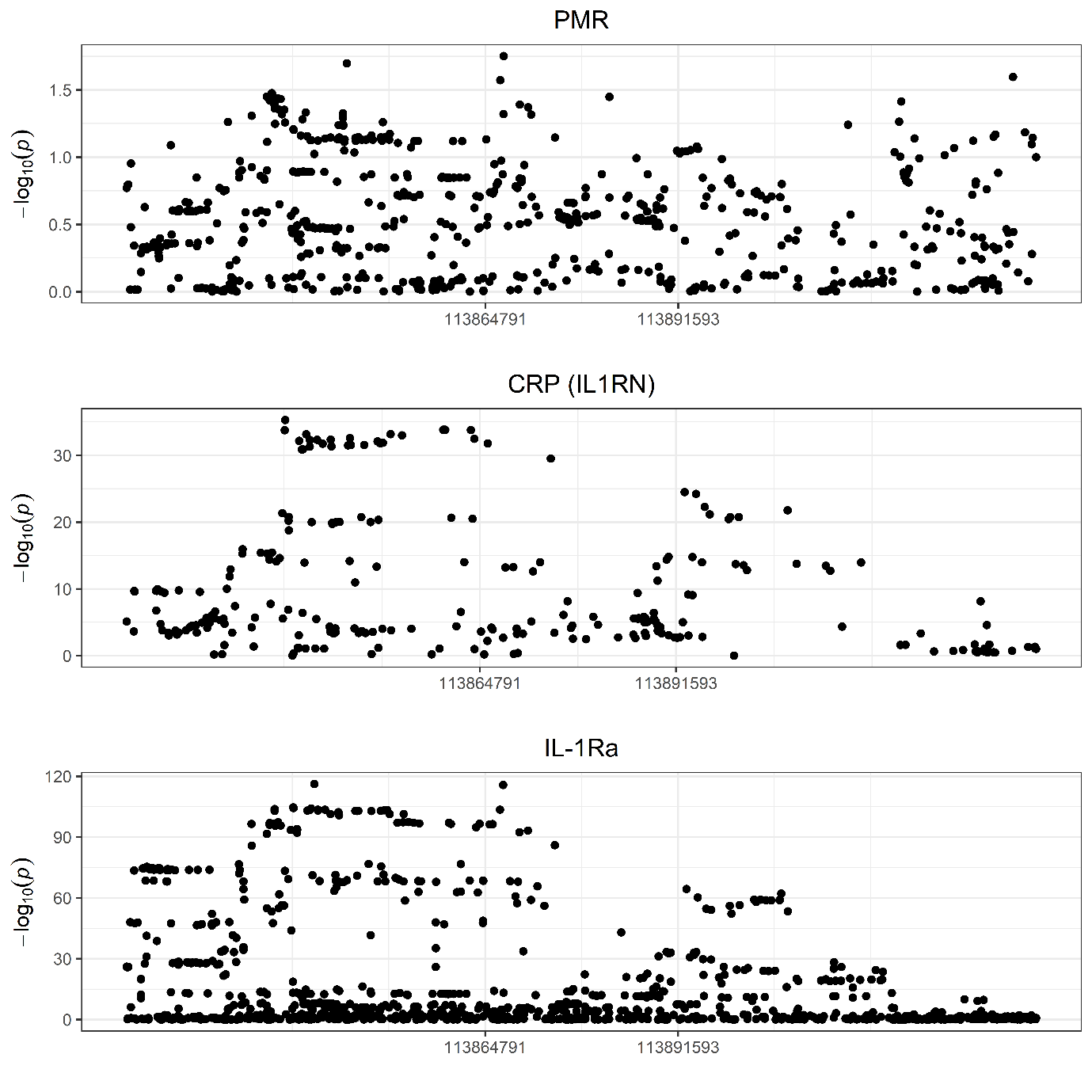


# Figure S2. Locus plots of PMR, CRP and IL-1Ra in and ± 50kilobases from the *IL1RN* gene region.

# Table S1. Single-nucleotide polymorphisms use to instrument IL-1Ra.

| Exposure | SNP | Base position (build GRCh37) on Chr2 | ea/oa | Exposure | | | | Outcome (PMR) | | | | F |
| --- | --- | --- | --- | --- | --- | --- | --- | --- | --- | --- | --- | --- |
|  |  |  |  | beta | se | eaf | p | beta | se | eaf | p |  |
| CRP | rs13409371 | 113838145 | A/G | 0.048 | 0.004 | 0.430 | 5.07E-36 | 0.046 | 0.025 | 0.408 | 0.063 | 58 |
| CRP | rs17624213 | 113933247 | T/G | 0.030 | 0.005 | 0.246 | 7.15E-09 | 0.006 | 0.028 | 0.273 | 0.818 | 44 |
| CRP | rs6743376 | 113832333 | A/C | 0.032 | 0.004 | 0.649 | 1.14E-16 | 0.040 | 0.026 | 0.654 | 0.119 | 35 |
| IL-1Ra | rs6743376 | 113832333 | A/C | -0.147 | 0.007 | 0.657 | 1.63E-86 | 0.040 | 0.026 | 0.654 | 0.119 | 58 |
| IL-1Ra | rs6734238 | 113841030 | A/G | 0.168 | 0.007 | 0.616 | 6.65E-117 | -0.041 | 0.025 | 0.586 | 0.095 | 131 |
| IL-1Ra | rs114189538 | 113821082 | A/G | -0.110 | 0.015 | 0.073 | 2.92E-14 | 0.088 | 0.051 | 0.067 | 0.081 | 81 |
| IL-1Ra | rs139076984 | 113839374 | T/G | 0.202 | 0.031 | 0.976 | 3.46E-11 | -0.191 | 0.098 | 0.981 | 0.052 | 41 |
| IL-1Ra | rs187780031 | 113841838 | T/G | 0.226 | 0.038 | 0.986 | 2.55E-09 | 0.103 | 0.107 | 0.988 | 0.334 | 526 |
| IL-1Ra | rs4252041 | 113890610 | T/C | -0.191 | 0.021 | 0.043 | 1.96E-19 | 0.014 | 0.057 | 0.048 | 0.809 | 394 |
| IL-1Ra | rs4849160 | 113934085 | A/G | -0.053 | 0.008 | 0.316 | 1.89E-10 | 0.019 | 0.026 | 0.350 | 0.454 | 125 |
| IL-1Ra | rs72829856 | 113913287 | A/G | 0.148 | 0.013 | 0.095 | 5.42E-29 | -0.017 | 0.043 | 0.092 | 0.691 | 44 |
| IL-1Ra | rs74667587 | 113895079 | A/G | -0.109 | 0.010 | 0.186 | 1.59E-30 | -0.003 | 0.032 | 0.177 | 0.926 | 35 |
| SNP, single nucleotide polymorphism; ea, effect allele; oa, other allele; se, standard error; eaf, effect allele frequency; F, F statistic. | | | | | | | | | | | | |

# Table S2. Colocalization analysis results.

| Analysis | SNP1 | SNP2 | H0 | H1 | H2 | H3 | H4 | H4/(H3+H4) |  |
| --- | --- | --- | --- | --- | --- | --- | --- | --- | --- |
| CRP biomarker | unconditioned | unconditioned | 2.76E-29 | 9.33E-01 | 2.34E-31 | 7.85E-03 | 5.96E-02 | 88.4% |  |
| CRP biomarker | rs13409371 | unconditioned | 2.16E-32 | 9.34E-01 | 1.18E-34 | 5.05E-03 | 6.07E-02 | 92.3% |  |
| CRP biomarker | rs7570267 | unconditioned | 3.21E-29 | 9.33E-01 | 1.36E-31 | 3.89E-03 | 6.35E-02 | 94.2% |  |
| Circulating IL-1Ra | unconditioned | unconditioned | 1.58E-109 | 9.28E-01 | 4.33E-111 | 2.53E-02 | 4.69E-02 | 64.9% |  |
| Circulating IL-1Ra | rs2441376 | unconditioned | 1.80E-109 | 9.43E-01 | 2.06E-111 | 1.08E-02 | 4.58E-02 | 81.0% |  |
| Circulating IL-1Ra | rs3811057 | unconditioned | 1.80E-109 | 9.44E-01 | 1.97E-111 | 1.03E-02 | 4.59E-02 | 81.7% |  |
| Circulating IL-1Ra | rs6734238 | unconditioned | 5.93E-82 | 9.43E-01 | 7.07E-84 | 1.12E-02 | 4.58E-02 | 80.4% |  |
| Posterior probability for  H0: neither trait has a genetic association in the region;  H1: only trait 1 has a genetic association in the region;  H2: only trait 2 has a genetic association in the region;  H3: both traits are associated, but with different causal variants;  H4: both traits are associated and share a single causal variant.  H4/(H3+H4) represents the probability of colocalization conditional on the presence of a causal variant for the outcome | | | | | | | | | |

# References

1. Davey Smith G, Hemani G. Mendelian randomization: genetic anchors for causal inference in epidemiological studies. Hum Mol Genet 2014;23:R89-98.

2. Burgess S, Zuber V, Valdes-Marquez E, Sun BB, Hopewell JC. Mendelian randomization with fine-mapped genetic data: Choosing from large numbers of correlated instrumental variables. Genet Epidemiol 2017;41:714–25.

3. Yavorska OO, Burgess S. MendelianRandomization: an R package for performing Mendelian randomization analyses using summarized data. International Journal of Epidemiology 2017;46:1734–9.

4. Robinson JW, Hemani G, Babaei MS, Huang Y, Baird DA, Tsai EA, et al. An efficient and robust tool for colocalisation: Pair-wise Conditional and Colocalisation (PWCoCo) [Internet]. 2022 [cited 2023 Jun 16];2022.08.08.503158. Available from: https://www.biorxiv.org/content/10.1101/2022.08.08.503158v1
